# Steepest near‐infrared spectroscopy‐derived deoxygenation slopes during arterial occlusions provide more reliable assessments of muscle mitochondrial capacity

**DOI:** 10.1113/EP093040

**Published:** 2025-12-12

**Authors:** Guillaume Costalat, Benoît Sautillet, Grégoire P. Millet, Clément Unal, Abd‐Elbasset Abaïdia, Abdellah Hassar, Maryne Cozette

**Affiliations:** ^1^ Faculty of Sport Sciences, APERE laboratory, UR‐UPJV 3300 University of Picardie Jules Verne Amiens France; ^2^ Institute of Sport Sciences University of Lausanne Lausanne Switzerland; ^3^ Department of Kinesiology Laval University Quebec Canada

**Keywords:** near‐infrared spectroscopy, oxidative metabolism, reliability, skeletal muscle

## Abstract

Assessment of near‐infrared spectroscopy (NIRS)‐derived muscle oxidative capacity relies on analysing deoxygenation slopes from NIRS signal versus time curves during brief arterial occlusions, which reflect the rate of post‐exercise recovery of muscle oxygen consumption (${\dot{V}}_{O_{2}m}$). However, current guidelines lack recommendations on the optimal selection of slopes for reliable measurement. The aim of the study was to compare a standardised partial‐segment approach against the conventional whole‐segment approach on the measurement and reliability of in vivo muscle oxidative capacity. Within the same session, 19 athletes (*n* = 9 sprinters; *n* = 10 middle‐distance runners) completed two NIRS‐derived muscle oxidative capacity trials on the vastus lateralis. Rate constants (*k*, min^−1^) were computed using the steepest ($k_{\mathrm{high}.S}$), whole ($k_{\mathrm{whole}.S}$) or shallowest ($k_{\mathrm{low}.S}$) deoxygenation slope from deoxyhaemoglobin (HHb) and muscle O_2_ saturation ($S_{mO_{2}}$) signals. Test–retest reliability [(coefficient of variation (CV), intraclass correlation coefficient (ICC)] and minimum difference (MD) were assessed. For the HHb signal, ICC analysis revealed moderate to excellent test–retest reliability for $k_{\mathrm{high}.S}$ [0.80 (0.54–0.92)], whereas poor to good reliability was observed for $k_{\mathrm{whole}.S}$ [0.71 (0.38–0.89)] and $k_{\mathrm{low}.S}$ [0.60 (0.19–0.83)]. $k_{\mathrm{high}.S}$ led to lower MD compared to $k_{\mathrm{whole}.S}$ and $k_{\mathrm{low}.S}$ (0.64 vs. 1.12 vs. 1.54 min^−1^, respectively). All three approaches led to significantly greater *k* values in runners compared to sprinters ($k_{\mathrm{high}.S}$:+32.2%, *P* < 0.001; $k_{\mathrm{whole}.S}$:+ 40.1%, *P* = 0.025; $k_{\mathrm{low}.S}$:+49.6%, *P* = 0.001). Compared to the conventional whole‐segment approach, selecting the steepest intra‐occlusion slope improved the reliability and sensitivity of NIRS‐derived mitochondrial capacity, likely by better reflecting instantaneous changes in ${\dot{V}}_{O_{2}m}$.

## Abstract (highlights)

- **What is the central question of this study?**
  To what extent does the selection of deoxygenation slopes during post‐exercise arterial occlusions affect the measurement and reliability of in vivo muscle oxidative capacity (*k*)?
- **What is the main finding and its importance?**
  Standardizing selection on the steepest intra‐occlusion slope yielded more reliable and sensitive estimates of the recovery constant *k*. This finding partly stems from a better ability of the steepest‐slope approach to capture instantaneous changes in ${\dot{V}}_{O_{2}m}$ than that of the conventional whole‐segment approach.

## 1 INTRODUCTION

Skeletal muscle oxidative capacity is a broad concept encompassing the assessment of mitochondrial function and is defined as the maximum rate of energy transfer to ATP through oxidative phosphorylation (Kemp et al., 2015). Traditionally, in vivo oxidative capacity has been evaluated by means of phosphorus magnetic resonance spectroscopy (^31^P‐MRS), based on the underlying assumption that post‐exercise phosphocreatine resynthesis is closely tied to oxidative metabolism (Arnold et al., 1984; Haseler et al., 1999; McMahon & Jenkins, 2002; Sahlin et al., 1979). More recently, in vivo investigation of skeletal muscle oxidative capacity through near‐infrared spectroscopy (NIRS) has gained interest due to its relative ease of use, portability and cost‐effectiveness compared to ^31^P‐MRS (Adami & Rossiter, 2018). Originating from an idea first proposed by Hamaoka and colleagues (Hamaoka et al., 1996; Motobe et al., 2004), NIRS‐derived mitochondrial capacity has shown significant correlations with gold standard ^31^P‐MRS (Ryan et al., 2013b) and well‐established laboratory techniques, such as mitochondrial protein content (Tripp et al., 2024) or mitochondrial respiration (Pilotto et al., 2022; Ryan et al., 2014). The NIRS‐based technique relies on active or electrically induced muscle contraction (Pelka et al., 2023; Ryan et al., 2013a, 2013b) followed by repeated, brief, intermittent arterial occlusions during which the linear change in deoxyhaemoglobin (HHb) or O_2_ muscle saturation ($S_{mO_{2}}$) is measured to estimate muscle O_2_ consumption (${\dot{V}}_{O_{2}m}$). Assuming a context where mitochondrial respiratory pathways achieve maximal activation (Chung et al., 2018), the decline in post‐exercise ${\dot{V}}_{O_{2}m}$ exhibits a first‐order exponential time course, allowing the calculation of a unique rate constant (*k*) that is proportional to muscle oxidative capacity (Adami & Rossiter, 2018).

The molecular cascade initiated by exercise training induces a series of adaptations that positively reshape the mitochondrial phenotype (Granata et al., 2018), thereby contributing to improvements in overall performance (Batterson et al., 2020; Jacobs et al., 2013). Consistent with these adaptations, numerous cross‐sectional studies have shown that NIRS‐derived mitochondrial capacity can distinguish populations with different levels of aerobic fitness (Adami et al., 2017, 2020; Brizendine et al., 2013; Erickson et al., 2013, 2015; Lagerwaard et al., 2021, 2019; Rasica et al., 2024; Villanova et al., 2025), supporting NIRS as a relevant technique for assessing longitudinal change or interventional efficacy in response to exercise training (Adami & Rossiter, 2018; Bellinger et al., 2020; Jeffries et al., 2018; Willingham & McCully, 2017). Taken together, these studies provide in vivo evidence of the extensive plasticity of muscle oxidative capacity, as reflected by maximal difference in *k* values, which can vary by more than six‐fold between diseased individuals and elite endurance athletes (Willingham & McCully, 2017).

Statistically speaking, high reliability is often essential to ensure a technique can detect true changes (Shechtman, 2013). Several factors, including sex, training background and age, may influence the reliability of a given test (Currell & Jeukendrup, 2008). NIRS‐derived oxidative capacity showed test–retest (same day) and day‐to‐day reliability, expressed as the coefficient of variation, ranging from 3.8% to 14.7%, and 8.4% to 43.5%, respectively (Adami et al., 2017, 2020; Bellinger et al., 2020; de Aguiar et al., 2022; Hanna et al., 2021; Jeffries et al., 2018; Ryan et al., 2012, 2013b; Zuccarelli et al., 2020). Beyond inherent within‐day/across‐day physiological variability, technical sources of variation (i.e., sampling error) may also contribute to measurement error (Shechtman, 2013). Consequently, recommendations have been established to ensure that variability due to measurement error is minimised: (i) averaging two or more consecutive trials to improve the signal‐to‐noise ratio (Adami & Rossiter, 2018; Barstow, 2019); (ii) adjusting for blood volume changes to ensure reciprocal changes in oxyhaemoglobin and HHb signals (Ryan et al., 2012); (iii) performing a ‘physiological calibration’ to limit the impact of adipose tissue thickness on NIRS signals (Ryan et al., 2012); and (iv) when voluntary contractions are used, standardising exercise intensity (e.g., as a percentage of maximal voluntary contraction) to account for its significant influence on ${\dot{V}}_{O_{2}m}$ recovery kinetics (Tripp et al., 2024).

To ensure this NIRS‐based technique provides an accurate estimate of oxidative capacity, it also needs to reliably capture instantaneous changes in muscle ${\dot{V}}_{O_{2}m}$ under non‐O_2_‐limiting conditions (Adami & Rossiter, 2018), whilst ensuring the signal‐to‐noise ratio is maximised throughout recovery (Ryan et al., 2013b). To date, numerous studies have adopted the protocol by Ryan *et al.*, considering the whole occlusion period as the analysis window to compute each post‐exercise ${\dot{V}}_{O_{2}m}$ (e.g., a 5‐s slope extracted from a 5‐s occlusion) (Ryan et al., 2012). Importantly, during our pre‐experiments (unpublished data), intra‐occlusion fluctuations in the NIRS signal were observed (illustrated in the second figure), questioning whether the whole‐segment approach provides the most accurate temporal resolution for estimating instantaneous changes in ${\dot{V}}_{O_{2}m}$. More recently, alternative strategies have emerged, including slope analyses over shorter windows within the occlusion (e.g., a 3‐s slope extracted from a ∼5‐s occlusion) (Beever et al., 2020; Zuccarelli et al., 2020), as well as oxygenation‐driven approaches in which the occlusion is guided by a target range of muscle oxygenation (e.g., 50–60% of physiological normalisation) instead of a fixed occlusion time (Pilotto et al., 2022; Villanova et al., 2025). In practice, slope selection in those approaches is either manual (Beever et al., 2020) or not explicitly defined (Pilotto et al., 2022), making the outcome potentially operator‐dependent and less reliable (Chung et al., 2018). Conversely, implementing a dedicated script to standardise slope selection by means of robust criteria could minimise operator‐related variability, thereby improving the robustness of the rate constant *k*.

This study aimed to determine whether slope selection based on a standardised partial‐segment approach improves the measurement and reliability of muscle mitochondrial capacity in a fit and healthy population. To address this, we assumed that for each post‐exercise occlusion, one may determine ‘three’ primary deoxygenation slopes: the steepest and the shallowest, representing the highest and lowest intra‐occlusion ${\dot{V}}_{O_{2}m}$, respectively, as well as the whole‐occlusion slope, covering the full occlusion period. The first two slopes were selected as they capture the range of intra‐occlusion changes in ${\dot{V}}_{O_{2}m}$, and were then compared against ${\dot{V}}_{O_{2}m}$ estimated over the whole occlusion period. Hence, the rate constant *k* was determined based on the highest ${\dot{V}}_{O_{2}m}$ (i.e., the steepest slope; $k_{\mathrm{high}.S}$), the lowest ${\dot{V}}_{O_{2}m}$ (i.e., the shallowest slope; $k_{\mathrm{low}.S}$), or using the whole occlusion period (i.e., the whole slope; $k_{\mathrm{whole}.S}$). We tested the hypothesis that consistently selecting the steepest slope would be the best approach, as it could better reflect the instantaneous recovery dynamic of ${\dot{V}}_{O_{2}m}$ whilst theoretically offering a stronger signal‐to‐noise ratio than the two other slopes. In practical terms, this was expected to translate into better test–retest reliability for $k_{\mathrm{high}.S}$ compared with $k_{\mathrm{whole}.S}$ and $k_{\mathrm{low}.S}$. Furthermore, we evaluated whether these three approaches yield interchangeable outcomes, an important consideration when selecting the most appropriate analysis for longitudinal monitoring or inter‐group comparisons. Finally, the study cohort consisted of trained athletes, specifically sprinters and middle‐distance runners. Given their distinct training regimens (Haugen et al., 2021), it was expected that runners would exhibit significantly greater *k* values than sprinters. Consequently, a second, more application‐oriented objective was to evaluate whether $k_{\mathrm{high}.S}$ would be more relevant than $k_{\mathrm{whole}.S}$ or $k_{\mathrm{low}.S}$ for capturing group differences in muscle mitochondrial capacity.

## 2 METHODS

### 2.1 Ethical approval

Each participant provided written informed consent after being fully informed of the experimental procedures. All procedures conformed to the standards set by the *Declaration of Helsinki*, except for registration in a database, and received local ethical approval (2025‐02‐1/2025‐50).

### 2.2 Participants

Nineteen healthy, male, physically active athletes (age: 21.1 ± 2.51 years; body mass index: 20.7 ± 1.5 kg m^−2^, relative body fat mass: 6.0 ± 2.6%) were recruited. For the sake of the second objective, the cohort was also split into two groups based on their sport discipline: sprinters (*n* = 9) and middle‐distance runners (*n* = 10). Sprinters were defined as athletes competing in races between 100 and 400 m, whilst middle‐distance runners included those competing at distances from 800 to 1500 m. All participants trained for at least 10 h per week and competed at a regional or national level. They were asked to refrain from heavy exercise 24 h prior to the start of the experiment.

### 2.3 Experimental design

**FIGURE 1:**
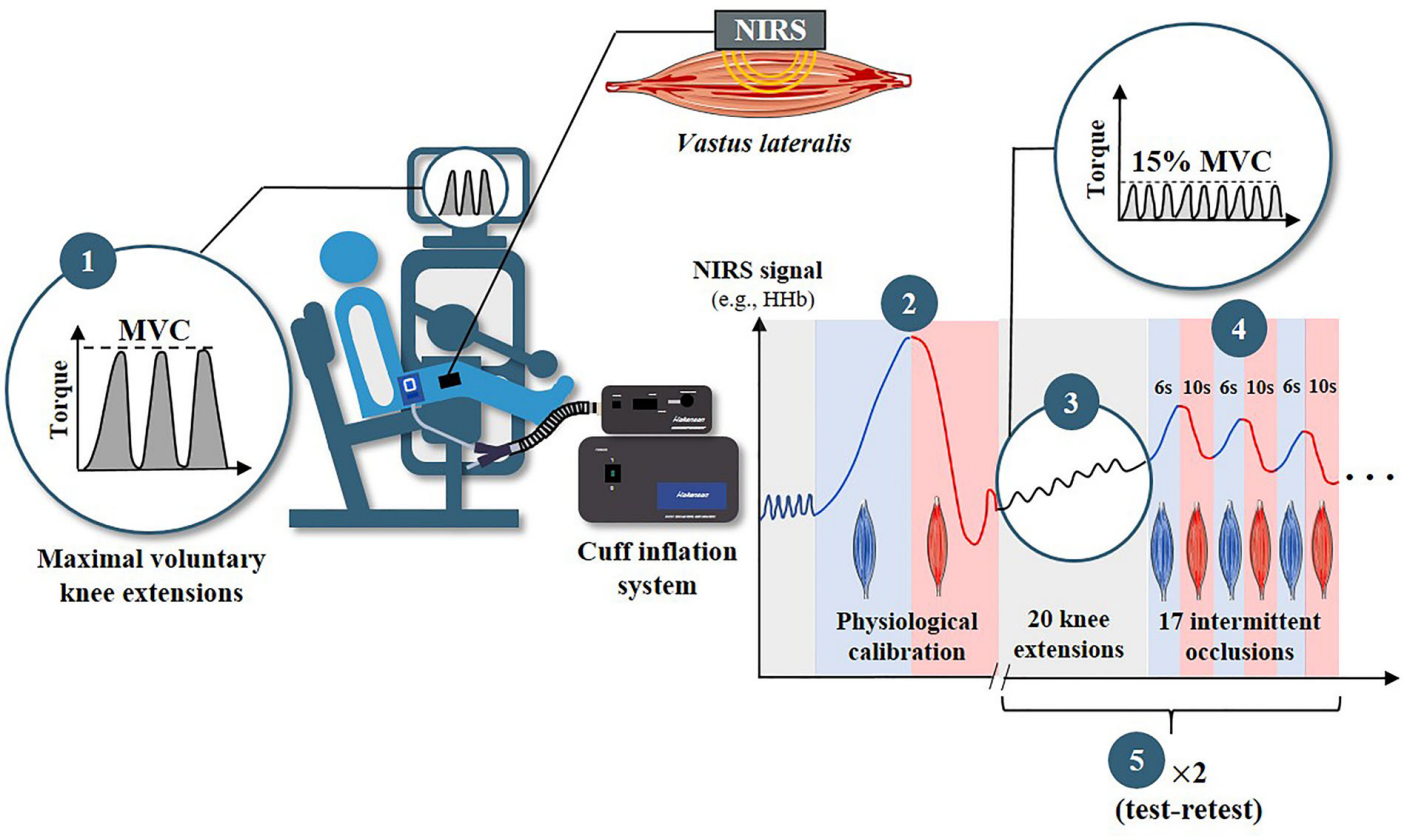
The cross‐sectional experimental design, depicting the key steps of the protocol. (1) Isokinetic maximal voluntary contraction (MVC) of the knee extensors (dominant leg) was assessed in sprinters (*n* = 9) and middle‐distance runners (*n* = 10). (2) Once equipped with a rapid cuff inflation system and NIRS probe positioned on the vastus lateralis, a physiological calibration (∼5 min arterial occlusion at 250 mmHg) was performed to identify the zenith and nadir values of the NIRS signal. (3) Athletes completed 20 isokinetic knee extensions at 15% of their individual's MVC. (4) The 17 intermittent occlusions (6 s on, 10 s off) were applied to estimate the recovery of muscle oxygen consumption. (5) Following a short recovery period (∼5 min), athletes completed a second trial, aimed at investigating test–retest reliability.

The study was designed to characterise intra‐occlusion variations in ${\dot{V}}_{O_{2}m}$ and assess how slope‐selection strategies (steepest, shallowest or based on the whole occlusion) influence the measurement, reliability and sensitivity of NIRS‐derived muscle mitochondrial capacity in a cohort of trained athletes. The key steps of the experimental design are summarised in Figure 1. First, athletes’ anthropometric data (body weight, height and fat mass) as well as their training history were collected. Following a familiarisation procedure, the mitochondrial capacity of the vastus lateralis (dominant leg) was assessed for each athlete. Once the NIRS signal returned to baseline values (∼ 5 min), a second trial was performed on the same leg to assess test–retest reliability. During the same visit, adipose tissue thickness (ATT) was measured at the site of NIRS interrogation using a skinfold caliper (Harpenden, Burges Hill, UK), assuming that the fat's layer was half of the skinfold measurement (Craig et al., 2017).

### 2.4 Isokinetic maximal voluntary contraction

NIRS‐derived mitochondrial capacity was assessed using a single‐joint exercise performed at a relative intensity corresponding to 15% of each individual's maximal voluntary contraction (MVC) (Beever et al., 2020). MVC was measured by means of an isokinetic dynamometer (Cybex Norm, division of Lumex, Inc., Ronkonkoma, NY, USA). Individuals were seated in an upright position and then fastened using straps to secure the chest, hip and ankle of the dominant (working) leg. The range of motion was set from 0° (full knee extension) to 90° knee flexion for all individuals. After an initial warm‐up, athletes completed two sets of three maximal concentric knee extensions at an angular velocity of 90° s^−1^, with the highest recorded peak torque value (MVC) used to set the intensity for evaluating vastus lateralis mitochondrial capacity.

### 2.5 Protocol to assess muscle oxidative capacity

With the same dynamometer setup used for MVC determination, individuals were equipped with a portable, wireless, continuous‐wave NIRS system (PortaMon, Artinis Medical Systems BV, Zetten, Netherlands) and a blood pressure cuff. The NIRS probe was positioned over the distal part of the vastus lateralis muscle belly, approximately 10–15 cm above the proximal border of the patella and 3–5 cm lateral to the midline of the thigh. The device was then attached using elastic bandages and wrapped in a black light‐absorbing cloth to prevent probe movement and contamination from extraneous light. To transiently occlude blood flow, a blood pressure cuff (SC12D) – connected to a rapid cuff inflation system (E20/AG101, Hokanson, Bellevue, Inc., WA, USA) – was positioned proximal from the NIRS probe.

The NIRS device emits three couples of near‐infrared lights (∼760 and ∼850 nm), each located at three standardised distances from the receiver (30, 35 and 40 mm). Lights at these two wavelengths are sensitive to changes in oxy‐ (HbO_2_) and deoxyhaemoglobin (HHb) concentrations. The contribution of both oxygenated and deoxygenated myoglobin to the NIRS signal accounts for more than 50% of the NIRS signal in mammalian muscles, including those of the human leg (Davis & Barstow, 2013). Consequently, HHb and HbO_2_ will hereafter refer to the combined concentrations of haemoglobin and myoglobin. Since light sources are in a spatially resolved configuration, an ‘absolute’ measure of muscle oxygen saturation ($S_{mO_{2}}$, %) can also be derived, representing the weighted average of arterial, capillary and venous blood oxygenation in relation to the total amount of haemoglobin (tHb, i.e., the sum of HbO_2_ and HHb). In the absence of arterial or venous occlusion, HbO_2_, HHb or $S_{mO_{2}}$ represent the dynamic balance between O_2_ supply and consumption. However, when combined with arterial occlusion, as in the present study, the resulting deoxygenation slopes provide estimates of ${\dot{V}}_{O_{2}m}$. In the present study, only HHb and $S_{mO_{2}}$ signals were considered for further analysis, as these are the most frequently reported variables within the literature (Rasica et al., 2024). NIRS signals were recorded at a sampling rate of 10 Hz, using a fixed differential pathlength factor (DPF) of 4 to account for photons scattering into the tissue. As each of the three NIRS channels provides an HHb signal, values were averaged to improve the signal‐to‐noise ratio.

**FIGURE 2:**
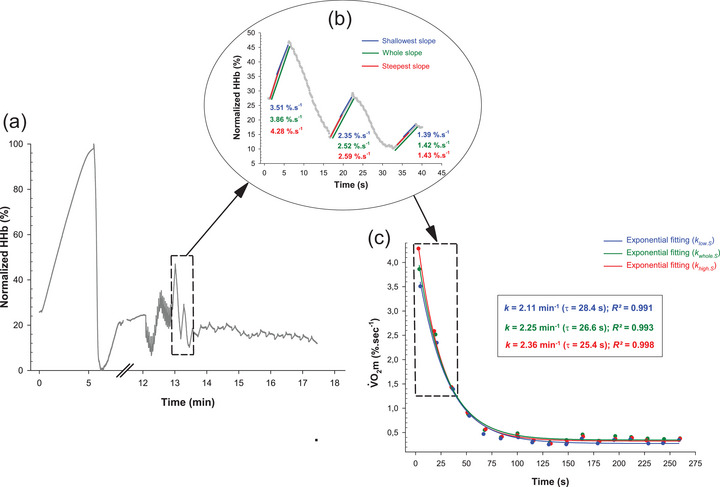
The methodology used to compute rate constants in a representative sprinter. (a) Kinetics of normalised HHb, corrected for blood volume changes, in a subject (only trial 1 is shown for clarity). (b) Computation of the highest (red slopes), whole‐occlusion (green slopes) and lowest (blue slopes) ${\dot{V}}_{O_{2}m}$ for each of the 17 arterial occlusions, using a custom MATLAB script (only occlusions 1–3 shown for clarity). (c) Exponential decay fittings applied to extract time (τ) and rate constants (*k*), either from the steepest (red data points, $k_{\mathrm{high}.S}$), whole‐occlusion (green data points, $k_{\mathrm{whole}.S}$) or shallowest (blue data points, $k_{\mathrm{low}.S}$) deoxygenation slopes. Note the excellent goodness‐of‐fit (*R* ^2^ > 0.99) for both whole‐ and partial‐segment approaches. The same methodology was used to compute rate constants from $S_{mO_{2}}$ signal.

Once the subjects were equipped and positioned on the isokinetic device, each testing session started with an ∼5‐min arterial occlusion (physiological calibration, 250 mmHg) to fully deoxygenate the muscle tissue at the site of NIRS interrogation (Figure 2a). After the cuff was released – allowing for the hyperaemic response and the signal to stabilise (∼3–5 min) – athletes performed a single set of 20 isokinetic knee extensions (15% MVC). Athletes were assisted with real‐time visual feedback on a computer screen to help them maintain the target exercise intensity. Seventeen brief arterial occlusions (6 s each at 250 mmHg), interspersed with 10 s intervals of cuff release, were administered over 4.5 min immediately following the exercise bout. Two measures of muscle oxidative capacity were performed consecutively (test–retest), with the second trial starting when both HHb and $S_{mO_{2}}$ signals had stabilised.

### 2.6 Blood volume change correction

Raw NIRS signals were first exported on a custom Microsoft Excel spreadsheet to compute the initial analysis. More precisely, the HHb signal, but not $S_{mO_{2}}$, was corrected for changes in blood volume at any given time ($\mathrm{HH}b_{c\;(t)}$) to ensure reciprocal changes between HbO_2_ and HHb throughout arterial occlusions (Ryan et al., 2012):

$$\mathrm{HH}b_{c\left(t\right)}=\mathrm{HH}b_{\left(t\right)}-\left(\mathrm{tH}b_{\left(t\right)}\cdot \beta_{\left(t\right)}\right)$$

where $\beta_{\left(t\right)}$, a correction factor is calculated at any given time (*t*), as follows:

$$\beta_{\left(t\right)}=\frac{\left|\mathrm{Hb}O_{2\left(t\right)}\right|}{\left|\mathrm{Hb}O_{2\left(t\right)}\right|+\left|\mathrm{HH}b_{\left(t\right)}\right|}$$

$S_{mO_{2}}$ is a variable that is already mathematically normalised to tHb and therefore theoretically does not require blood volume correction (Adami & Rossiter, 2018). Finally, the HHb_c_ signal was normalised on a 0–100% scale, with 100% defined by the zenith value during physiological calibration (i.e., the 5‐min arterial occlusion) and 0% by the nadir value upon release of the blood pressure cuff (Figure 2a). The same normalisation procedure was used for $S_{mO_{2}}$.

### 2.7 Partial‐segment approach: algorithm for slopes detection

Subsequently, normalised signals (in % s^−1^, Figure 2a) were exported in a custom MATLAB (MathWorks, Natick, MA, USA) script designed to eliminate operator influence in calculating the 17 slopes of the HHb (and $S_{mO_{2}}$) versus time curves. For each 6 s arterial occlusion, the steepest and the shallowest slope – determined over a 3 s span of data (i.e., 30 consecutive data points) – were selected to compute $k_{\mathrm{high}.S}$ and $k_{\mathrm{low}.S}$, respectively (Figure 2b, c). Briefly, the script initially identified all 3‐s monotonic linear segments within the dataset, selecting positive (increasing) segments for HHb and negative (decreasing) segments for $S_{mO_{2}}$, considering a specified fit quality criterion (minimum *R* ^2^ threshold) that could be adjusted by the operator. A linear segment was considered as increasing (HHb signal) or decreasing ($S_{mO_{2}}$ signal) whenever 30 consecutive moving first‐derivative calculations – each computed from three consecutive data points – yielded positive or negative values, respectively. Segments were constrained to a fixed duration of 3 s. Therefore, if no sequence of 30 consecutive increasing (HHb signal) or decreasing ($S_{mO_{2}}$ signal) data points was detected, the corresponding occlusion was ignored. The steepest and shallowest slopes could overlap within the same occlusion, as shown in Figure 2b. At this stage, several hundred linear segments were detected for a given trial. To solve this issue, a parameter setting representing the expected elapsed time between distinct arterial occlusions was implemented (usually set to 3 s), allowing the selection of a single segment (either the steepest or the shallowest) for each of the 17 arterial occlusions. To ensure consistency, *R* ^2^ threshold was set at 0.99 for all analyses, meaning that any linear segment displaying a *R* ^2^ < 0.99 was ignored. This restrictive threshold was chosen to ensure high‐quality slope fitting whilst avoiding the detection of spurious slopes (artefacts) that are observed immediately after cuff occlusion or release. When the script was unable to detect the 17 post‐exercises ${\dot{V}}_{O_{2}m}$ (i.e., the 17 linear slopes), the raw normalised NIRS signal was pre‐processed with a moving average filter, initially set to the minimum window size of three points (i.e., 0.3 s). If required, the window size was steadily increased until all potentially detectable slopes were identified. In our view, this stepwise increase in the window size was the best compromise to detect enough slopes for accurate curve fitting whilst minimising the impact of the filter on the data. To ensure the robustness of the script, a co‐author of the present study (B.S.) independently duplicated the slope analysis manually. The *k* values obtained from both approaches (automated vs. manual) were highly consistent, regardless of whether the steepest or shallowest slopes were considered, confirming the accuracy of the script.

### 2.8 Whole‐segment approach: algorithm for slopes detection

To assess the potential benefit of the partial‐segment approach, we compared it with the common method, namely the whole‐slope approach, where each deoxygenation slope is calculated across the full occlusion range (∼6 s). This procedure was also partly automated with a MATLAB script. Briefly, the script first detected local maxima and minima every 16 s (the occlusion period + the subsequent release phase) to define the full occlusion segment. For the numerous slopes in which artifacts were present (systematically located near the segment boundaries), a secondary manual verification was carried out to adjust the start and end points of the regression, whilst ensuring that the duration never exceeded 6 s. Unlike the partial‐segment approach, no minimum *R* ^2^ criterion for linear slope regression was imposed in the whole‐slope analysis, since the ‘entire’ occlusion was used to compute the linear regression. Finally, the signal was not pre‐processed (i.e., filtered) to mirror current practice.

### 2.9 Computation of *k* (muscle mitochondrial capacity)

Slopes (i.e., ${\dot{V}}_{O_{2}m}$) were then plotted against time and fitted with a first‐order exponential decay to compute muscle mitochondrial capacity, as follows (Figure 2c):

$${\dot{V}}_{O_{2}m}\left(t\right)={\dot{V}}_{O_{2}\mathrm{mb}}+A_{\dot{V}O_{2}m}\cdot \mathrm{ex}p^{\left(\frac{-t}{\tau }\right)}$$

Where ${\dot{V}}_{O_{2}m}$ (t) is the ${\dot{V}}_{O_{2}m}$ at any given time *t* (%.s^−1^); ${\dot{V}}_{O_{2}\mathrm{mb}}$ is the basal (steady‐state) ${\dot{V}}_{O_{2}m}$ (% s^−1^); $\;A_{\overset{\cdot }{V}O_{2}m}$ is the amplitude of the exponential decay (% s^−1^) and; τ is the time constant of the exponential decay (s).

Finally, the time constant τ was converted into a rate constant *k* $\left[k\;\left(\mathrm{mi}n^{-1}\right)=\frac{1}{\tau \;(\min)}\right]$, so that this final output can be interpreted as proportional to muscle mitochondrial capacity. Rate constants *k* were calculated from HHb and $S_{mO_{2}}$ signals. For each of these two signals, *k* values were determined either based on the highest ($k_{\mathrm{high}.S}$), whole‐occlusion ($k_{\mathrm{high}.S}$) or the lowest ($k_{\mathrm{low}.S}$) intra‐occlusion ${\dot{V}}_{O_{2}m}$, resulting in a total of six *k* values per individual.

The *k* values from the two trials were averaged to give an overall measure of muscle oxidative capacity, which was then used to compare sprinters and middle‐distance runners. The quality control of *k* computation was checked based on three criteria: (i) the number of slopes utilised for *k* computation; (ii) the coefficient of determination (*R* ^2^) to assess the goodness of the exponential fit; and (iii) the maximal fold‐change in ${\dot{V}}_{O_{2}m}$ above baseline to confirm that the exercise stimulus activated mitochondrial respiratory pathways (Hanna et al., 2021). Maximal fold‐change was estimated as ${\dot{V}}_{O_{2}m}$ from the first post‐exercise occlusion divided by the mean ${\dot{V}}_{O_{2}m}$ of the last five occlusions (asymptotic part of the exponential curve, referred to as ‘baseline’).

### 2.10 Changes in ${\dot{V}}_{O_{2}m}$ and $S_{mO_{2}}$ within occlusions

For each of the 17 arterial occlusions, both the highest and lowest intra‐occlusion deoxygenation slopes were determined, as detailed earlier in the manuscript (Figure 2). To elucidate whether the partial‐segment approach better reflects ${\dot{V}}_{O_{2}m}$ dynamics than the whole‐segment approach, the time points at which the steepest ($\mathrm{Tim}e_{\mathrm{high},S}$) and the shallowest slope ($\mathrm{Tim}e_{\mathrm{low},S})$ occurred were recorded for each occlusion. The time difference (ΔTime) was then calculated by subtracting $\mathrm{Tim}e_{\mathrm{low},S}$ with $\mathrm{Tim}e_{\mathrm{high},S}$ for both HHb and $S_{mO_{2}}$ signals (panels (a, c) of the seventh figure). A significant positive value, indicating that the shallowest slope was detected after the steepest one, was interpreted as evidence that the behaviour of the intra‐occlusion NIRS signal is not strictly linear. Conversely, if no clear pattern in the occurrence of ${\dot{V}}_{O_{2}m}$ was detected (i.e., mean ΔTime = 0), it was interpreted as evidence that the fluctuations in the intra‐occlusion NIRS signal stemmed primarily from random measurement error. In addition, within each occlusion, the maximal intra‐occlusion variation in ${\dot{V}}_{O_{2}m}$ ($\Delta {\dot{V}}_{O_{2}m}$) was quantified by subtracting ${\dot{V}}_{O_{2}m}$ corresponding to the shallowest slope (${\dot{V}}_{O_{2}m,\mathrm{low},S}$) with ${\dot{V}}_{O_{2}m}$ corresponding to the steepest slope (${\dot{V}}_{O_{2}m,\mathrm{high},S}$) (panels (b, d) of the seventh figure).

### 2.11 Statistical analysis

A prospective power analysis was conducted to calculate the required sample size (G*Power software version 3.1). The primary outcome variable of interest was the change in muscle mitochondrial capacity (sprinters vs. middle‐distance runners) in a two‐way within‐between measures design, with an anticipated small effect size (*f* = 0.15) and high correlation between repeated measurements (*r* = 0.85). To maintain a type I error rate of 0.05 whilst ensuring a statistical power of 0.80, the present study required a sample size of 20 individuals. This calculation ensured an 80% likelihood of detecting the expected effect size, given the actual level of significance.

The samples were first tested for normality with the Shapiro–Wilk test. For changes in mitochondrial capacity, two‐way within‐between ANOVA was conducted to investigate the effect of analytical approach (HHb $k_{\mathrm{high}.S}$ vs. HHb $k_{\mathrm{low}.S}$ vs. HHb $k_{\mathrm{whole}.S}$ vs. $S_{mO_{2}}$ $k_{\mathrm{high}.S}$ vs. $S_{mO_{2}}$ $k_{\mathrm{whole}.S}$ vs. $S_{mO_{2}}$ $k_{\mathrm{low}.S}$), group (sprinters vs. middle‐distance runners) and their interaction (analytical approach × group). A one‐way repeated measures ANOVA was used for other variables, such as ΔTime, Δ${\dot{V}}_{O_{2}m}$ and fitting quality criteria. Prior to conducting ANOVA, the assumption of sphericity was checked with Mauchly's test. If Mauchly's test was significant, the degrees of freedom were corrected using Greehouse–Geisser estimates of sphericity. Multiple pairwise comparisons adjusted with the Bonferroni correction were conducted whenever the main effect or interaction was significant.

Relative reliability (i.e., between‐subject variability) was assessed using the intraclass correlation coefficient (ICC, a.u.) for both $k_{\mathrm{high}.S}$ and $k_{\mathrm{low}.S}$. Specifically, the ICC_(3,1)_, a two‐way mixed‐effects model with absolute agreement, was used. This approach accounts for fixed raters and random subjects (Koo & Li, 2016), making it suitable for evaluating single measurements per rater as in the present study. ICC < 0.5, 0.5 ≤ ICC < 0.75, 0.75 ≤ ICC < 0.90 and ICC ≥ 0.90 were considered to indicate poor, moderate, good and excellent reliability, respectively (Koo & Li, 2016). ICC estimates were compared to the threshold indicative of ‘moderate reliability’ (i.e., 0.5) by means of a one‐sample *F*‐test. Absolute reliability (i.e., within‐subject reliability) was quantified through the typical error (TE), determined as the standard deviation of the differences between the two repeated measurements divided by √2. TE was then converted into a coefficient of variation (CV, %), providing a percentage of the within‐subject variability. In addition, the precision of individual scores (i.e., *k* values) was estimated using the standard error of measurement (SEM), calculated as follows (Weir, 2005):

$$\mathrm{SEM}=\mathrm{SD}\cdot \sqrt{1-\mathrm{IC}C_{\left(3,1\right)}}$$

where SEM is the standard error of measurement for *k* (min^−1^); SD is the standard deviation of the scores *k* from all subjects (min^−1^) and; ICC_(3,1)_ is the intraclass coefficient previously calculated (a.u.).

Finally, the SEM was used to determine the minimum difference (MD) to be considered practically meaningful and is calculated as follows (Weir, 2005):

$$\mathrm{MD}=\mathrm{SEM}\cdot 1.96\cdot \sqrt{2}$$

where MD is the minimum difference for *k* (min^−1^).

To assess the agreement between the two approaches (i.e., $k_{\mathrm{high}.S}$ vs. $k_{\mathrm{low}.S}$), Bland–Altman plots were used. Systematic bias was tested using a one‐sample *t*‐test comparing the mean difference to zero, and the 95% limits of agreement (LoA) were calculated as the mean bias ± 1.96 × SD of the differences. Statistical analysis was performed and graphs created using standard R packages (RStudio team, 2024) and Sigma Plot software (version 14.5, Systat Software, San Jose, CA, USA), respectively. Statistical significance for all analyses was accepted at *P *< 0.05.

## 3 RESULTS

Data from one subject did not meet our methodological criteria for slope detection, likely due to an error in the pressure cuff setup. As a result, nine sprinters and 10 middle‐distance runners were included in the statistical analysis, representing a total of 114 rate constants *k* analysed. Fifteen out of 19 athletes reached a consistent plateau in the NIRS signal by the end of the physiological calibration.

### 3.1 Muscle $S_{mO_{2}}$ during single‐leg exercise and brief arterial occlusions

**FIGURE 3:**
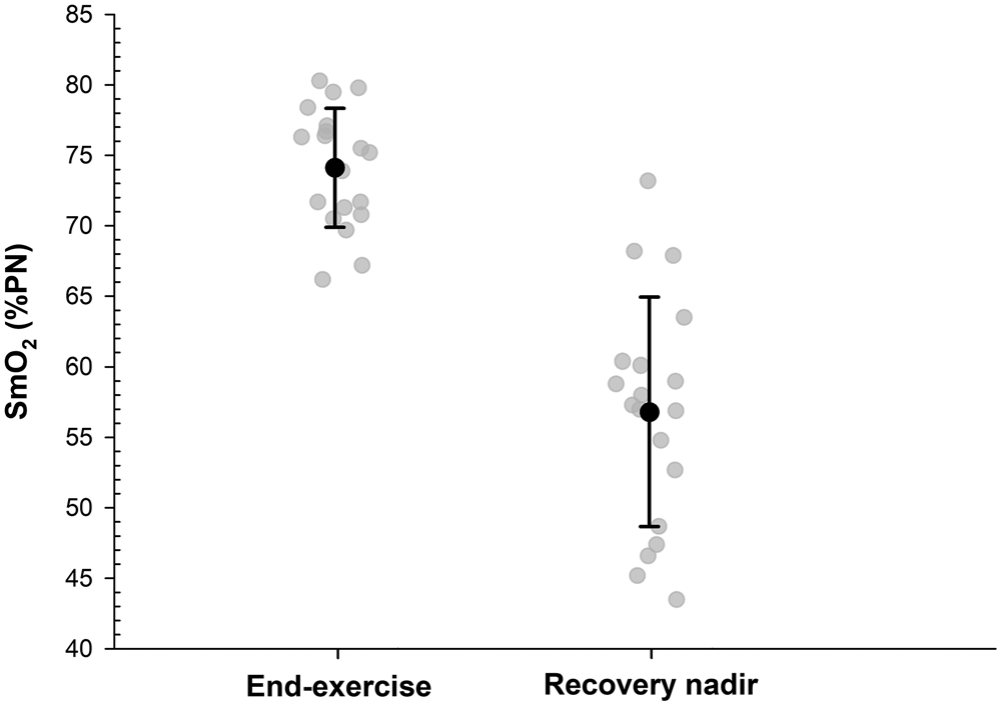
Individual and mean (±SD) values of normalised $S_{mO_{2}}$ at end‐exercise and at recovery nadir.

Normalised $S_{mO_{2}}$ values at the end of exercise and recovery nadir are presented in Figure 3. At the end of the 20 isokinetic knee extensions, the mean normalised $S_{mO_{2}}$ (relative to the individual physiological range) was 74.1 ± 4.2% in all athletes. Specifically, no difference was observed between the first and second trials (73.7 ± 4.5% vs. 74.6 ± 4.7%, respectively; *P* = 0.303), nor between sprinters and middle‐distance runners (73.7 ± 3.9% vs. 74.4 ± 4.6%, respectively; *P *= 0.727).

The nadir normalised $S_{mO_{2}}$ during repeated occlusions was 56.8 ± 8.1% in all athletes. Specifically, no difference was observed between the first and second trials (57.3 ± 9.1% vs. 56.3 ± 7.9%, respectively; *P* = 0.405), nor between sprinters and middle‐distance runners (55.6 ± 7.6% vs. 57.9 ± 8.8%, respectively; *P* = 0.539*)*.

### 3.2 Computation of the rate constant *k*: quality criteria

Quality criteria for *k* computation are summarised in Table 1. To maximise slope detection without compromising our methodological standard (i.e., *R* ^2^ > 0.99, see ‘Methods’), the $S_{mO_{2}}$ signal required a slightly stronger filtering of raw data compared to the HHb signal (all *P* < 0.05). For the HHb signal, the number of slopes detected to compute *k* was similar between the three approaches (all *P* > 0.05), whilst this number was slightly higher for $k_{\mathrm{whole}.S}$ compared to $k_{\mathrm{high}.S}$ and $k_{\mathrm{low}.S}$ for $S_{mO_{2}}$ signal (all *P* < 0.05). For the HHb signal, the quality of adjustment of the exponential fitting (*R* ^2^) was higher for $k_{\mathrm{high}.S}$ compared to $k_{\mathrm{whole}.S}$ (*P* = 0.042) and $k_{\mathrm{low}.S}$ (*P* = 0.012), whilst no difference was observed for the $S_{mO_{2}}$ signal (all *P* > 0.05). The maximal increase in ${\dot{V}}_{O_{2}m}$ following exercise was consistent across methodological approaches (*F*_(2.0, 35.4)_ = 3.0; *P* = 0.061, $\omega_{p}$ ^2^ = 0.017). At the site of NIRS interrogation, the mean ATT across all athletes was 3.4 ± 1.1 mm and no difference in ATT was observed between sprinters and middle‐distance runners (3.4 ± 1.2 vs. 3.3 ± 1.1 mm, respectively; *P* = 0.81).

**TABLE 1:** Mean (±SD) quality control criteria associated to the measurement of muscle mitochondrial capacity.

	HHb			$S_{mO_{2}}$		
	*k*_high,S_	*k*_whole,S_	*k*_low,S_	*k*_high,S_	*k*_whole,S_	*k*_low,S_
Moving average filtering (s)	0.13 ± 0.14	—	0.13 ± 0.14	0.84 ± 0.36^#^	—	0.84 ± 0.36^#^
Number of slopes (out of 17)	16.7 ± 0.7	16.9 ± 0.3	16.7 ± 0.7	15.3 ± 1.4*	16.8 ± 0.5	15.1 ± 1.4*
Mono‐exponential fit (*R* ^2^)	0.99 ± 0.01^†^	0.97 ± 0.04	0.96 ± 0.06	0.99 ± 0.01	0.99 ± 0.01	0.99 ± 0.00
${\dot{V}}_{O_{2}m}$: maximal fold‐change above baseline	7.7 ± 3.2	7.3 ± 3.1	7.1 ± 3.0	8.5 ± 3.0	7.3 ± 3.1	7.9 ± 2.3

# *P* < 0.05: significant difference between $S_{mO_{2}}$ and HHb signals. ^*^ *P *< 0.05: for $S_{mO_{2}}$ signal, significant difference with $k_{\mathrm{whole},S}.$ ^†^ *P* < 0.05: for HHb signal, significant difference with $k_{\mathrm{whole}.S}$ and $k_{\mathrm{low},S}.$ See text for abbreviations.

### 3.3 Agreement of $k_{\mathrm{high}.S}$ and $k_{\mathrm{low},S}\;$ with the whole‐segment approach

**FIGURE 4:**
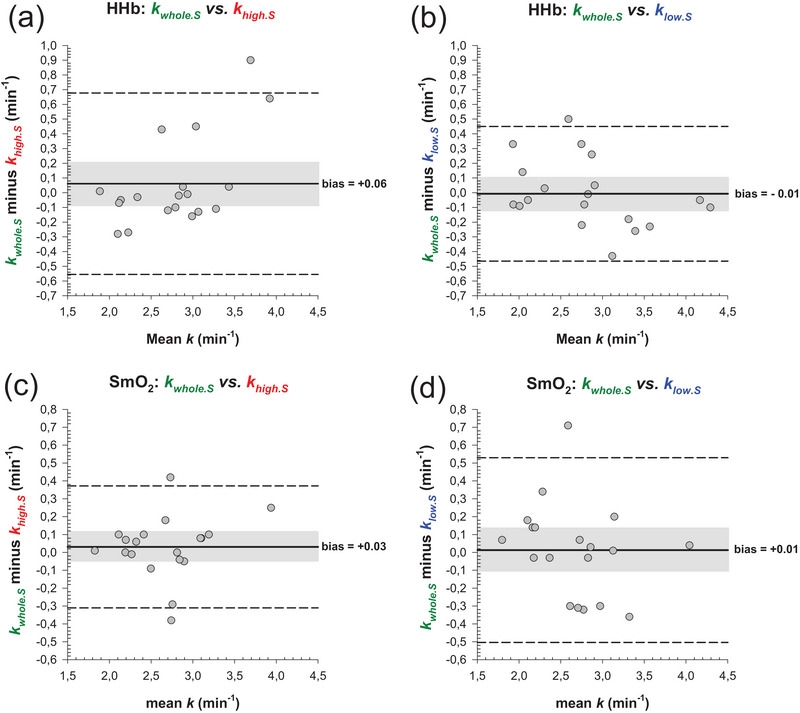
Bland–Altman plots illustrating the mean difference and 95% limits of agreement (LoA) between $k_{\mathrm{high}.S}$ and $\;k_{\mathrm{whole},S}$, and between $k_{\mathrm{low}.S}$ and $\;k_{\mathrm{whole},S}$ (*n* = 19) for HHb signal (a, b) and $S_{mO_{2}}$ signal (c, d). The continuous black line represents the mean difference and the grey shaded area denotes the 95% confidence interval of the mean difference. Note that no systematic bias was observed between $k_{\mathrm{high}.S}$ and $\;k_{\mathrm{whole},S}$, nor between $k_{\mathrm{low}.S}$ and $\;k_{\mathrm{whole},S}$.

Agreements of $\;k_{\mathrm{high},S}$ and $k_{\mathrm{low}.S}$ with $\;k_{\mathrm{whole},S}$ are displayed in Figure 4a–d. For HHb signal, no systemic bias was observed between $\;k_{\mathrm{whole},S}$ and $k_{\mathrm{high}.S}$ (mean difference: 0.06 min^−1^; *P* = 0.408) nor between $k_{\mathrm{whole}.S}$ and $k_{\mathrm{low}.S}$ (mean difference: 0.01 min^−1^; *P* = 0.892). Similarly, regarding $S_{mO_{2}}$ signal, no systematic bias was observed between $k_{\mathrm{whole}.S}$ and $k_{\mathrm{high}.S}$ (mean difference: 0.03 min^−1^; *P* = 0.447) nor between $k_{\mathrm{whole}.S}$ and $k_{\mathrm{low}.S}$ (mean difference: 0.01 min^−1^; *P* = 0.83).

### 3.4 Test‐retest reliability: $k_{\mathrm{high}.S}$ versus $k_{\mathrm{whole}.S}$ versus $k_{\mathrm{low}.S}$

**FIGURE 5:**
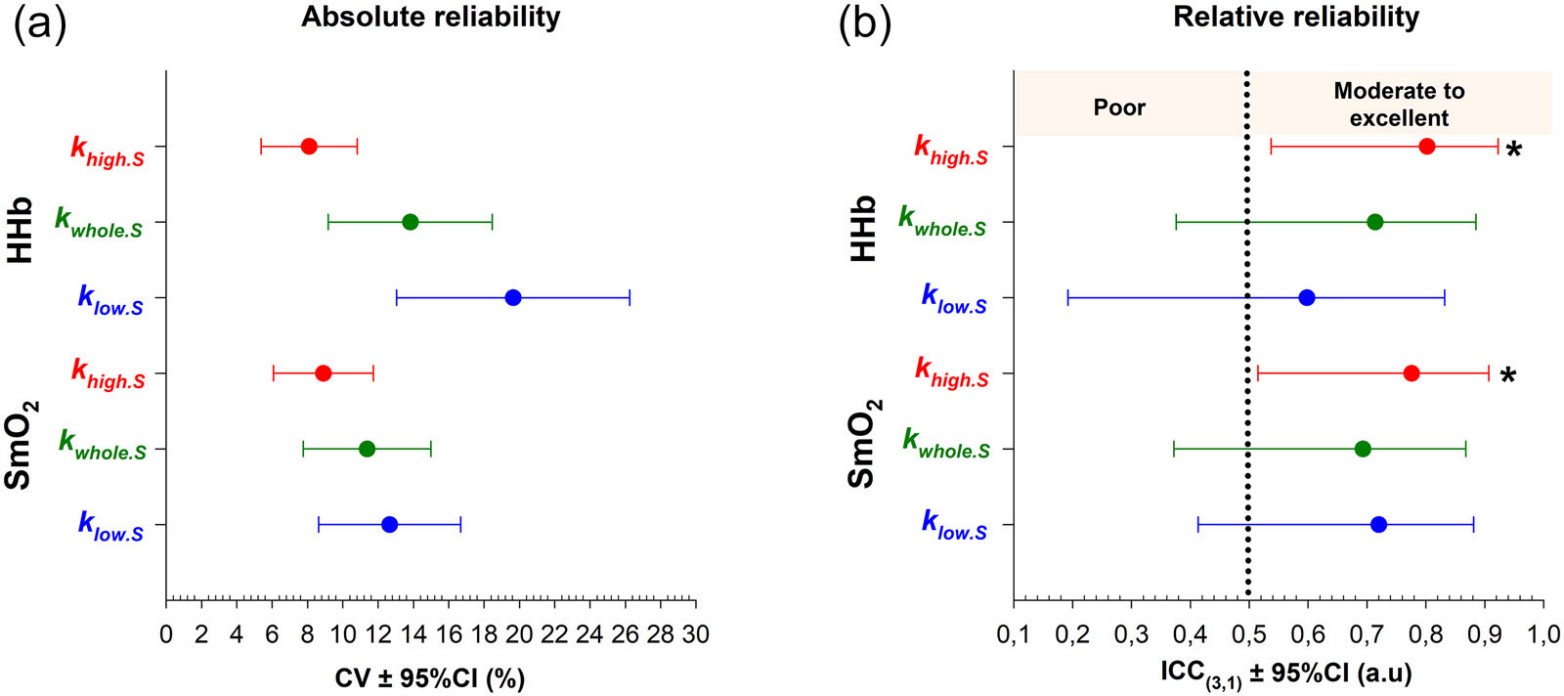
For each NIRS signal, the reliability for $k_{\mathrm{high}.S}$, $k_{\mathrm{whole}.S}$ and $k_{\mathrm{low}.S}$ in all athletes (*n* = 19). (a) Absolute test–retest reliability (±95% CI). (b) Relative test–retest reliability (±95% CI). *P < 0.05 indicates a significant difference between ICC estimates and the ICC value of 0.5, the threshold distinguishing ‘poor’ from ‘moderate reliability’.

For each NIRS signal and analytical approach, test–retest absolute (CV) and relative reliability (ICC_3,1_) of *k* values is presented in Figure 5a, b. CVs were consistently lower for $k_{\mathrm{high}.S}$ compared to $k_{\mathrm{whole}.S}$ and $k_{\mathrm{low}.S}$, regardless of the NIRS signal investigated (Figure 5a). For $k_{\mathrm{high}.S}$, ICC estimate (95% CI) was 0.80 (0.54–0.92) for HHb and 0.78 (0.52–0.91) for $S_{mO_{2}}$, indicating moderate to excellent reliability in both NIRS signals (Figure 5b). For $k_{\mathrm{whole}.S}$, ICC estimate (95% CI) was 0.71 (0.38–0.89) for HHb and 0.69 (0.37–0.87) for $S_{mO_{2}}$, reflecting poor to good reliability in both NIRS signals (Figure 5b). Similarly, for $k_{\mathrm{low}.S}$, ICC estimate (95% CI) was 0.60 (0.19–0.83) for HHb and 0.72 (0.41–0.88) for $S_{mO_{2}}$, reflecting poor to good reliability in both NIRS signals (Figure 5b).

### 3.5 Oxidative capacity in sprinters versus runners: $k_{\mathrm{high}.S}$ versus $k_{\mathrm{whole}.S}$ versus $k_{\mathrm{low}.S}$

**FIGURE 6:**
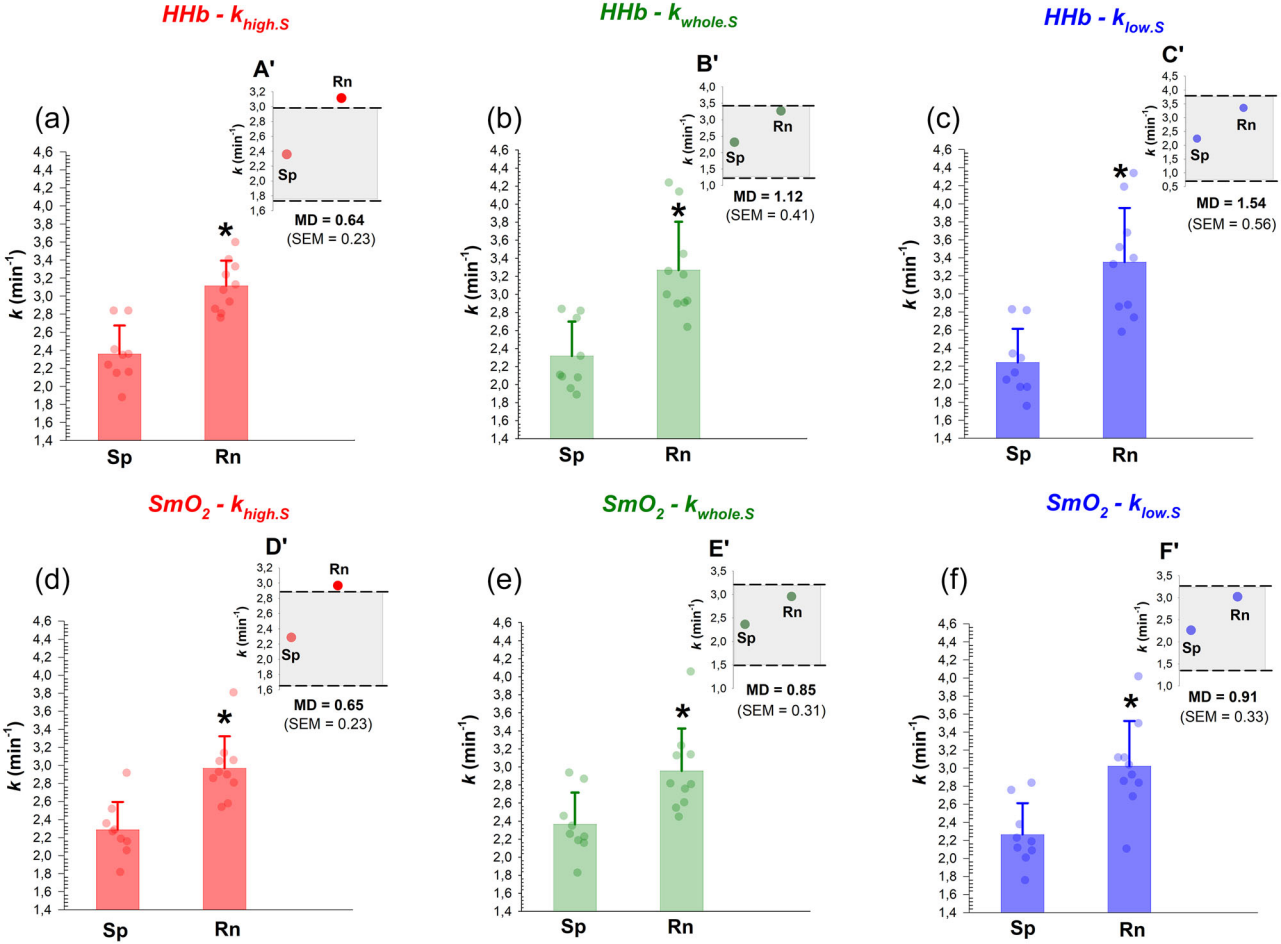
Individual data points and mean (± SD) rate constants between sprinters (Sp, *n* = 9) and middle‐distance runners (Rn, *n* = 10). (a) $k_{\mathrm{high}.S}$ derived from the HHb signal. (b) $k_{\mathrm{whole}.S}$ derived from the HHb signal. (c) $k_{\mathrm{low}.S}$ derived from the HHb signal. (d) $k_{\mathrm{high}.S}$ derived from $S_{mO_{2}}$ signal. (e) $k_{\mathrm{whole}.S}$ derived from $S_{mO_{2}}$ signal. (f) $k_{\mathrm{low}.S}$ derived from $S_{mO_{2}}$ signal. Panels (a′–f′) illustrate the magnitude of the minimum difference (MD, grey area) with regards to the mean rate constant of the two cohorts. The standard error of measurement (SEM) used to compute MD is also reported for each method. Regardless of the NIRS signal investigated, note that changes in $k_{\mathrm{high}.S}$ exceeded the MD, but not for $k_{\mathrm{low}.S}$ nor $k_{\mathrm{whole}.S}$. *P < 0.05 indicates a significant difference between sprinters and middle‐distance runners.

For each NIRS signal, muscle mitochondrial capacity between the two cohorts, evaluated by means of the three approaches, is illustrated in Figure 6a–f. No significant effect was observed for analytical approach (*F*_(2.7, 45.1)_ = 2.2; *P* = 0.107, $\omega_{p}$ ^2^ = 0.016), whereas a significant group (*F*_(1, 17)_ = 23.3; *P* = 0.0002, $\omega_{p}$ ^2^ = 0.383) and interaction effect (*F*_(2.7, 45.1)_ = 3.5; *P* = 0.027, $\omega_{p}$ ^2^ = 0.032) was evident. For HHb, pairwise comparisons revealed that middle‐distance runners had greater $k_{\mathrm{high}.S}$ than sprinters (3.12 ± 0.28 vs. 2.36 ± 0.32 min^−1^, respectively; *P* < 0.001; Figure 6a), greater $k_{\mathrm{whole}.S}$ than sprinters (3.27 ± 0.54 vs. 2.32 ± 0.38 min^−1^, respectively; *P* = 0.025; Figure 6b) and greater $k_{\mathrm{low}.S}$ compared to sprinters (3.35 ± 0.60 vs. 2.24 ± 0.37 min^−1^, respectively; *P* = 0.001; Figure 6c). Similarly, for $S_{mO_{2}}$, middle‐distance runners had significantly greater $k_{\mathrm{high}.S}$ than sprinters (2.97 ± 0.35 vs. 2.29 ± 0.31 min^−1^, respectively; *P* = 0.002; Figure 6d), as well as significantly greater $k_{\mathrm{whole}.S}$ (2.96 ± 0.47 vs. 2.37 ± 0.35, respectively; *P* = 0.041; Figure 6e) and $k_{\mathrm{low}.S}$ (3.02 ± 0.50 vs. 2.26 ± 0.35 min^−1^, respectively; *P* = 0.009; Figure 6f) compared to sprinters.

### 3.6 Minimum difference (MD): $k_{\mathrm{high}.S}$ versus $k_{\mathrm{whole}.S}$ versus $k_{\mathrm{low}.S}$

For each NIRS signal, the measurement error expressed as the MD, is presented in Figure 6a′–f′. Regarding the HHb signal, $k_{\mathrm{high}.S}$ revealed a lower MD than that of $k_{whole.S}$ and $k_{\mathrm{low}.S}$ (0.64 vs. 1.12 vs. 1.54 min^−1^, respectively; Figure 6a′–c′). Similarly, for the $S_{mO_{2}}$ signal, the MD of $k_{\mathrm{high}.S}$ was lower than that of $k_{\mathrm{whole}.S}$ and $k_{\mathrm{low}.S}$ (0.65 vs. 0.85 vs. 0.91 min^−1^, respectively; Figure 6d′–f′).

### 3.7 Differences in the slope occurrence time (ΔTime) and maximal intra‐occlusion variation in ${\dot{V}}_{O_{2}m}$($\Delta {\dot{V}}_{O_{2}m}$)

**FIGURE 7:**
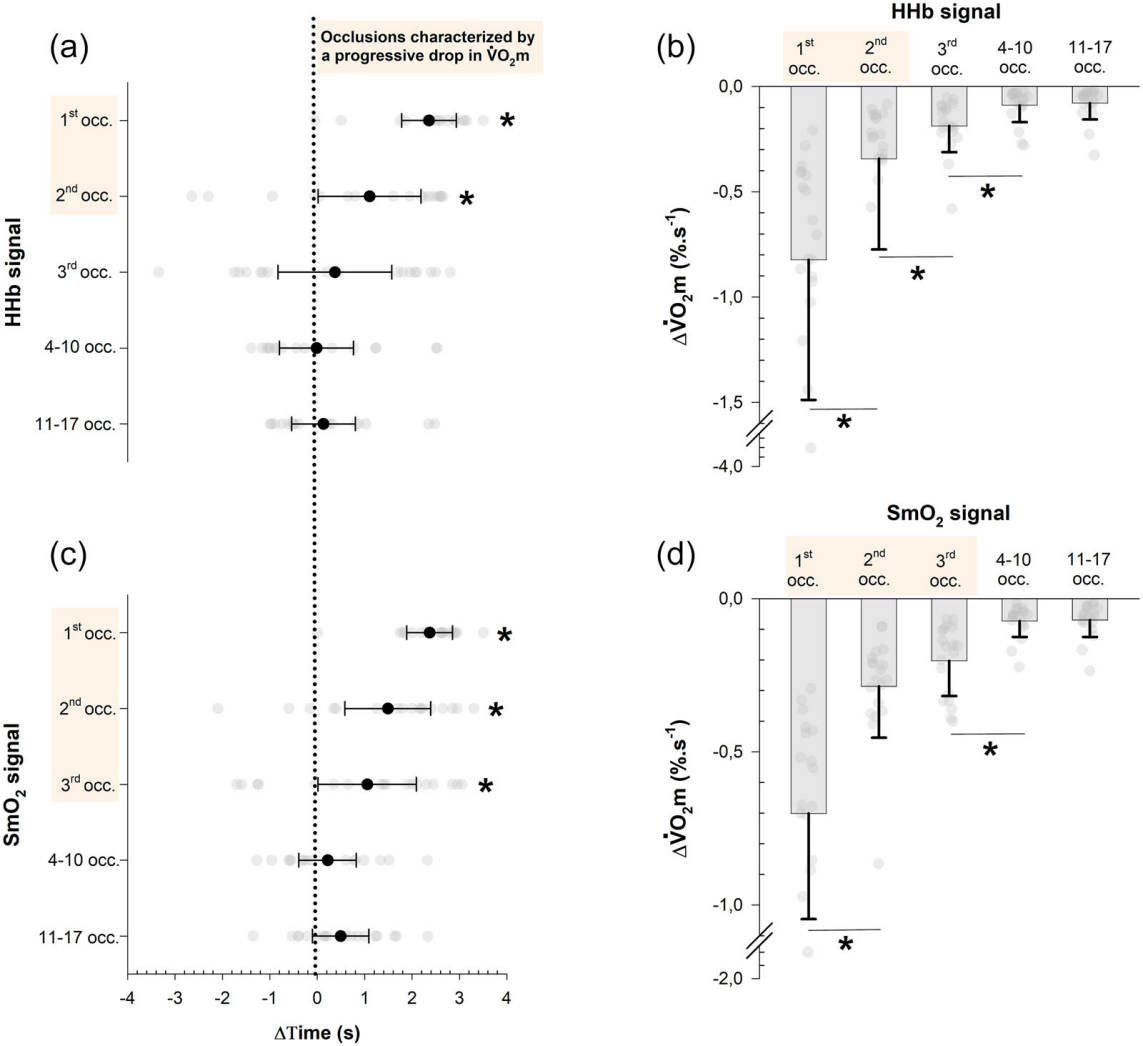
(a, c) Individual data points and mean (95% CI) elapsed time (ΔTime) between the occurrence of the lowest and the highest ${\dot{V}}_{O_{2}m}$. The orange shaded areas serve to emphasise occlusions where the shallowest deoxygenation slope occurred after the steepest one (positive ΔTime). Occ.: occlusion. *P < 0.05 indicates a significant positive difference from the reference line (0 value). (b, d) Individual data points and mean (±SD) maximal intra‐occlusion variation in ${\dot{V}}_{O_{2}m}$. The orange shaded areas serves to emphasise occlusions characterised by a progressive decrease in ${\dot{V}}_{O_{2}m}$ over the occlusion period.

The difference in the slope occurrence time (ΔTime) and the corresponding maximal intra‐occlusion variation in ${\dot{V}}_{O_{2}m}$ ($\Delta {\dot{V}}_{O_{2}m}$) are presented in Figure 7a–d. A significant effect of occlusion was found on ΔTime for both HHb (*F*_(2.5, 44.4)_ = 22.3; *P* < 0.001, ω^2^ = 0.284) and $S_{mO_{2}}$ signals (*F*_(2.4, 43.4)_ = 15.7; *P* < 0.001, ω^2^ = 0.294). Specifically, for the HHb signal, ΔTime was significantly greater than the reference value (0) during the first (+2.4 ± 0.9 s; *P* < 0.001) and second (+1.1 ± 1.64 s; *P* = 0.045) occlusion, but not during subsequent occlusions (all *P* = 1) (Figure 7a). Regarding the $S_{mO_{2}}$ signal, ΔTime was also significantly greater than the reference value during the first (+2.4 ± 0.7 s; *P* < 0.001), second (+1.5 ± 1.4 s; *P* < 0.001), and third (+1.1 ± 1.6 s; *P* = 0.046) occlusion, whilst no such difference was observed during subsequent occlusions (all *P* > 0.05) (Figure 7c).

There was a significant effect of occlusion on $\Delta {\dot{V}}_{O_{2}m}$ for HHb (*F*_(1.2, 21.0)_ = 21.8; *P* < 0.001, ω^2^ = 0.357) and $S_{mO_{2}}$ signals (*F*_(1.4, 25.9)_ = 52.0; *P* < 0.001, ω^2^ = 0.613). More precisely, $\Delta {\dot{V}}_{O_{2}m}$ was maximal during the first occlusion for HHb and $S_{mO_{2}}$, representing a relative decrease in ${\dot{V}}_{O_{2}m}$ of 20.6 ±9.5% for HHb and 18.8 ±6.8% for $S_{mO_{2}}$, before progressively declining across subsequent occlusions (Figure 7b, d).

### 3.8 Relative noise sensitivity

**FIGURE 8:**
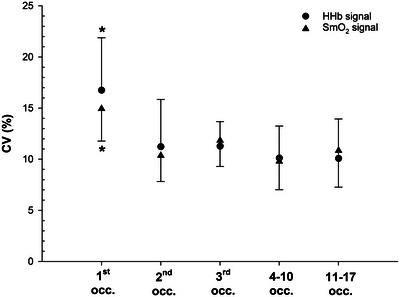
CV‐based noise sensitivity (±95% CI) across the 17 occlusions. *P < 0.05 indicates a significant difference between the first occlusion and the remaining ones. CV derived from steepest and shallowest slopes.

Two‐point CV proxy from the steepest and shallowest slopes are presented in Figure 8. A significant effect of occlusion was found on CV for both HHb (*F*_(1.9, 34.2)_ = 4.9; *P* = 0.015, ω^2^ = 0.087) and $S_{mO_{2}}$ signals (*F*_(2.7, 48.1)_ = 4.9; *P* = 0.009, ω^2^ = 0.073). For both signals, pairwise comparisons revealed that the CV from the first occlusion was significantly higher than the remaining occlusions (*all P* < 0.05).

## 4 DISCUSSION

Assuming that each post‐exercise occlusion comprises ‘three’ main deoxygenation slopes that can be used to estimate NIRS‐derived muscle oxidative capacity, the key findings of this study were as follows: (i) Whilst mean group analysis demonstrated that all three approaches discriminated sprinters from runners, ICC estimates revealed that both $k_{\mathrm{whole}.S}$ and $k_{\mathrm{low}.S}$ exhibited poor to good reliability, whereas $k_{\mathrm{high}.S}$ showed moderate to excellent reliability; (ii) consequently, $k_{\mathrm{high}.S}$ resulted in lower SEM and MD than $k_{\mathrm{whole}.S}$ or $k_{\mathrm{low}.S}$, indicating greater sensitivity of $k_{\mathrm{high}.S}$ to track individual changes in oxidative capacity; and (iii) the first two to three occlusions were consistently characterised by decreases in ${\dot{V}}_{O_{2}m}$ over the course of the occlusion time, revealing that the partial‐segment approach better captures the instantaneous change in ${\dot{V}}_{O_{2}m}$ compared to the conventional whole‐segment approach. Taken together, the present findings demonstrate that selecting the steepest deoxygenation slope ensures more consistent and sensitive estimates of muscle oxidative capacity, regardless of the NIRS signal investigated (i.e., HHb or $S_{mO_{2}}$).

Since the pioneering studies that aimed at assessing NIRS‐derived mitochondrial capacity (Hamaoka et al., 1996; Ryan et al., 2012), several modified versions have been proposed over the last decade (Beever et al., 2020; Dickinson et al., 2024; Hanna et al., 2021; Manferdelli et al., 2022; Pilotto et al., 2022; Sumner et al., 2020; Tripp et al., 2024; Zuccarelli et al., 2020), along with efforts to standardise procedures through the development of guidelines (Adami & Rossiter, 2018; Barstow, 2019). However, a notable limitation in the existing literature is the lack of standardised criteria to compute ${\dot{V}}_{O_{2}m}$. For the first time, the reliability of two standardised analytical approaches, designed to reduce operator influence in calculating post‐exercise ${\dot{V}}_{O_{2}m}$, was investigated and compared to the conventional whole‐segment approach. The major findings were that $k_{\mathrm{high}.S}$ had a lower CV and a higher ICC than $k_{\mathrm{whole}.S}$ and $k_{\mathrm{low}.S}$ (Figure 5), demonstrating greater absolute and relative reliability when the steepest deoxygenation slope was selected. Specifically, confidence interval analysis revealed that $k_{\mathrm{high}.S}$ had moderate to excellent relative reliability, whereas $k_{\mathrm{whole}.S}$ and $k_{\mathrm{low}.S}$ exhibited only poor to good reliability (Figure 5b) (Koo & Li, 2016). Notably, the reliability of $k_{\mathrm{high}.S}$ fell within the upper range reported in studies on test–retest reliability, whilst $k_{\mathrm{whole}.S}$ and $k_{\mathrm{low}.S}$ closely aligned with the lower range (Beever et al., 2020; de Aguiar et al., 2022; Tripp et al., 2024). Bland–Altman graphs revealed no systematic bias in *k* values between the partial‐segment and the whole‐segment approach (Figure 4). In addition, all three approaches demonstrated that middle‐distance runners had greater mitochondrial capacity in the vastus lateralis than sprinters (Figure 6), confirming that the NIRS technique detects between‐group (mean) differences in muscle oxidative capacity, even amongst healthy individuals (Brizendine et al., 2013; Villanova et al., 2025). However, group‐level inference does not take into account reliability and therefore cannot indicate which approach is best suited to detect meaningful individual change. Reliability analysis, therefore, acts as a complementary statistic, ensuring that variability due to measurement error is minimised and thus allowing true effects to be distinguished from noise (Weir, 2005). Random noise was calculated using the minimum difference (MD), a metric that quantifies the amount of change needed to confidently state that an observed difference exceeds the measurement error (Weir, 2005). In other words, the MD metric is an individual‐level threshold derived from test–retest error (i.e., the SEM) that reflects the boundaries of measurement error (Vaz et al., 2013). Notably, $k_{\mathrm{high}.S}$ led to a lower SEM than $k_{\mathrm{whole}.S}$ and $k_{\mathrm{low}.S}$, thus resulting in a lower MD (Figure 6). As a hypothetical example, if an athlete displays a gain in $k_{\mathrm{high}.S}$ equivalent to the mean group difference, for example, following a training intervention, this would be interpreted as a meaningful improvement in oxidative capacity since the difference exceeds the MD (Figure 6a′, d′). In contrast, this interpretation would not apply when using $k_{\mathrm{whole}.S}$ (Figure 6b′, e′) or $k_{\mathrm{low}.S}$ (Figure 6c′, f′). Taken together, these findings support $k_{\mathrm{high}.S}$ as the most sensitive approach for evaluating individual responses to any intervention designed to target mitochondrial adaptations.

Several NIRS‐based protocols assessing muscle mitochondrial capacity use slope windows shorter than the occlusion period, that is, partial‐segment analyses (Beever et al., 2020; Pilotto et al., 2022; Tripp et al., 2024; Zuccarelli et al., 2020), potentially leaving slope selection to operator discretion. For example, in the present study, approximately 30 linear segments of 3 s could be calculated for each arterial occlusion. Considering the number of occlusions, this would theoretically result in a very large number of distinct rate constants, corresponding to the number of possible linear slope combinations (∼30^17^). In addition, all exponential fits successfully converged and revealed high goodness‐of‐fit, regardless of whether the whole, steepest or shallowest deoxygenation slope was used (*R* ^2^ > 0.95, Table 1). Taken together, without a standardised procedure, suboptimal slope selection (i.e., below the steepest slope) is likely and, importantly, nearly undetectable, even for trained operators. This could partly explain the poor test–retest reliability of recovery *k* sometimes reported in the literature (Barstow, 2019).

For a given occlusion, the steepest and shallowest deoxygenation slopes physiologically correspond to the highest and lowest ${\dot{V}}_{O_{2}m}$, respectively. Within the first two (HHb signal) or three ($S_{mO_{2}}$ signal) occlusions, the lowest ${\dot{V}}_{O_{2}m}$ consistently occurred after the highest one, as evidenced by the significant positive time difference between $\mathrm{Tim}e_{\mathrm{low},S}$ and $\mathrm{Tim}e_{\mathrm{high},S}$ (i.e., ΔTime) only observed during initial occlusions (Figure 7a, c). This clear statistical trend demonstrates that ${\dot{V}}_{O_{2}m}$ progressively declined over the course of occlusion time (up to ∼20%, Figure 7b, d), and therefore strongly suggests that this phenomenon cannot be solely attributed to random measurement error. Such rapid decline was already noted in the pioneering work of Hamaoka and colleagues and likely motivated their choice to analyse ${\dot{V}}_{O_{2}m}$ over a relatively short time window (i.e., 3 s) (Hamaoka et al., 1996). Consistent with this, our results confirm that a 3‐s time window can detect the rapid decline in metabolic rate even during brief occlusions, indicating the NIRS signal is better described as biphasic than strictly linear over the full occlusion period. Accordingly, this demonstrates that partial‐segment analysis more effectively characterises instantaneous ${\dot{V}}_{O_{2}m}$ dynamics, especially in the early recovery phase, which may partly explain the higher reliability of $k_{\mathrm{high}.S}$ compared to the conventional whole‐segment approach. This is also supported by the small but significantly higher goodness‐of‐fit (*R* ^2^) observed for HHb $k_{\mathrm{high}.S}$ (Table 1). By contrast, no such difference in *R* ^2^ appeared for $S_{mO_{2}}$, likely because stronger smoothing (to ensure slopes *R* ^2^ > 0.99) flattened intra‐occlusion ${\dot{V}}_{O_{2}m}$ dynamics, thereby minimising differences between $S_{mO_{2}}$ $k_{\mathrm{high}.S}$ and $S_{mO_{2}}$ $k_{\mathrm{whole}.S}$ (Table 1). Beyond inherent decline in post‐exercise metabolic rate, intra‐occlusion changes in ${\dot{V}}_{O_{2}m}$ could also be explained by transient O_2_ mismatch due to ongoing O_2_ consumption without replenishment (trapped blood) (Tevald et al., 2009). However, our results do not support any hypoxic issue, as both end‐exercise $S_{mO_{2}}$ and the recovery nadir remained above ∼50% (Figure 3), well above the $S_{mO_{2}}$ range (0–20%) associated with O_2_ limitation and subsequent slope‐steepness alterations (Pilotto et al., 2022). For confirmation, further studies that experimentally modulate tissue O_2_ availability whilst computing $k_{\mathrm{high}.S}$ and $k_{\mathrm{low}.S}$ are warranted.

From slopes 3 to 17 (HHb signal) and 4 to 17 ($S_{mO_{2}}$ signal), approximately half of the shallowest slopes occurred before the steepest ones, and vice versa (Figure 6a, c). The absence of a clear pattern in slope occurrence implies that the order of occurrence is largely driven by random measurement noise rather than by any physiological event. Finally, to compare noise sensitivity across recovery, we estimated a two‐point CV proxy from the steepest and shallowest slope. CV peaked at the first occlusion (∼15–17%) and then stabilised (∼10%), indicating localised heteroscedasticity (Figure 8). Consequently, any source of noise occurring during the first occlusion will have a more impactful effect on *k* in both relative (Figure 8) and absolute terms (Figure 7b, d). In our young, healthy athletes, this concerns the first occlusion, but in frail or clinical populations – where rate constants are typically lower (Lagerwaard et al., 2020; Willingham & McCully, 2017) – the same issue could possibly extend to more occlusions due to a less pronounced steep phase of the exponential curve. For the remaining occlusions, the relative impact of noise remained constant (Figure 8), whilst its absolute influence progressively declined towards the asymptotic part of recovery (Figure 7b, d). Considering both our results and published observations, slope selection should be standardised to the steepest segment not only at the initial recovery point but at least all along the steep phase of the exponential decay.

### 4.1 Limitations

Standardising slope selection on the highest intra‐occlusion ${\dot{V}}_{O_{2}m}$ is likely the most relevant approach for estimating NIRS‐derived mitochondrial capacity. However, this finding is primarily based on an analysis of reliability. Whether $k_{\mathrm{high}.S}$ provides greater validity than $k_{\mathrm{low}.S}$ or any other non‐standardised slope selection approach remains to be established through comparisons with gold‐standard techniques, such as ^31^P‐MRS or direct assessment of mitochondrial respiration.

Several factors may confound the interpretation of ${\dot{V}}_{O_{2}m}$ recovery curves, one of which is ATT, which covers the underlying tissue of interest. In the present study, ATT at the site of NIRS interrogation ranged from 2.0 to 5.7 mm with no significant difference observed between the two cohorts of athletes. Although no universal ATT cut‐off value exists, previous works have suggested that ATT values up to 6–8 mm do not compromise the NIRS signal (Franceschini et al., 1998; Stuer et al., 2025). In addition, prior to *k* computation, the NIRS signal was systematically scaled to the individual maximal physiological range, a pre‐processing step which was shown to alleviate the influence of ATT on ${\dot{V}}_{O_{2}m}$ estimation (Ryan et al., 2012). Taken together, one may assume that ATT did not substantially influence ${\dot{V}}_{O_{2}m}$ recovery kinetics in the present study.

Finally, our sample size may appear relatively modest for a reliability study (*n* = 19). However, it covers a broad range of physiologically ‘healthy’ *k* values (∼2 to ∼4 min^−1^). Nevertheless, it remains to determine how the choice of deoxygenation slope would influence more vulnerable populations. Lower range of *k* values (<2 min^−1^), a hallmark of frail and/or clinical populations (Lagerwaard et al., 2020; Willingham & McCully, 2017), are presumably associated with impaired microvascular function (Tonson et al., 2017), implying that some of the recovery slopes could be affected by transient mismatch between local O_2_ availability and demand, making the choice of slope potentially even more clinically important.

### 4.2 Conclusion

By comparing three analytical approaches, the present study provides key methodological insights for optimising NIRS‐derived measurements of muscle oxidative capacity in healthy individuals. The main finding was that standardising slope detection to the steepest deoxygenation slope substantially improved the test–retest reliability of the recovery constant *k*. This outcome partly stems from a better ability of the steepest‐slope approach to capture instantaneous changes in ${\dot{V}}_{O_{2}m}$ than that of the conventional whole‐segment approach. Consequently, when monitoring individual responses to training intervention, suboptimal slope selection (i.e., below the steepest slope) could possibly mask true improvements in muscle oxidative capacity. Minimising the operator‐related variability by adopting a standardised slope selection procedure participates in maximising the interpretability of NIRS‐derived recovery *k* in both research and applied settings.

## AUTHOR CONTRIBUTIONS

Conceived and designed the research: Guillaume Costalat and Maryne Cozette. Performed experiments: Guillaume Costalat, Maryne Cozette, Benoît Sautillet and Clément Unal. Interpreted results of the experiment: Guillaume Costalat, Clément Unal, Abd‐Elbasset Abaïdia and Abdellah Hassar. Drafted the manuscript: Guillaume Costalat and Benoît Sautillet. Edited and revised the manuscript: Maryne Cozette, Grégoire P. Millet and Abd‐Elbasset Abaïdia. Approved the final version of the manuscript: Maryne Cozette and Grégoire P. Millet.

All authors have read and approved the final version of this manuscript and agree to be accountable for all aspects of the work in ensuring that questions related to the accuracy or integrity of any part of the work are appropriately investigated and resolved. All persons designated as authors qualify for authorship, and all those who qualify for authorship are listed.

## CONFLICT OF INTEREST

None declared.

## Data Availability

All data of the present study are available from the corresponding author upon reasonable request.
